# Residual metabolic burden in young psoriasis patients successfully treated with biologics

**DOI:** 10.1007/s00403-024-03403-4

**Published:** 2024-09-27

**Authors:** Eva Klara Merzel Šabović, Tadeja Kraner Šumenjak, Miodrag Janić

**Affiliations:** 1 Department of Dermatovenerology, University Medical Centre Ljubljana, Gradiškova ulica 10, Ljubljana, Slovenia; 2 Faculty of Medicine, University of Ljubljana, Vrazov trg 2, Ljubljana, Slovenia; 3 Faculty of Agriculture and Life Sciences, University of Maribor, Pivola 10, Hoče, Slovenia; 4 Clinical Department of Endocrinology, Diabetes and Metabolic Diseases, University Medical Centre Ljubljana, Zaloška 7, Ljubljana, Slovenia

**Keywords:** Psoriasis, Residual metabolic burden, Insulin resistance, Biologic therapy

## Abstract

Metabolic disorders are common in patients with psoriasis and contribute significantly to an increased cardiovascular risk. While biologic therapy is very successful in clearing skin lesions, its impact on metabolic parameters is uncertain. Our aim was to investigate the residual metabolic burden in psoriasis patients successfully treated with biologic therapy. We conducted a cross-sectional study of 80 young patients (54 men, 26 women, aged 30–45 years) successfully treated with either adalimumab, secukinumab or guselkumab and topical therapy or methotrexate, and 20 healthy controls. Anthropometric parameters, lipid levels and metabolic indices (HOMA-IR, TyG index and FIB-4 index) were measured. Patients did not receive any other treatments to exclude confounding effects. After analysis, we found that patients treated with three different biologics had similar metabolic status, only the FIB-4 index was higher in the adalimumab group than in the secukinumab and guselkumab treatment groups. There were no significant differences between the patients treated with biologics and the control group. The comparison with patients treated topically or with methotrexate showed that only triglyceride levels, HOMA-IR, TyG index, and FIB-4 index were elevated in patients treated with adalimumab compared to patients treated with topical therapy. Finally, metabolic status was also similar in patients treated with methotrexate or topical therapy. In conclusion, this study suggests that psoriasis patients successfully treated with biologics have similar metabolic parameters to the control group and patients treated with topical therapy or methotrexate. This indicates that there is no significant residual metabolic burden in young patients successfully treated with biologics. These results are clinically relevant and should be considered in the treatment of psoriasis patients.

The study is registered at http://clinicaltrials.gov (identifier: NCT05957120). Date of registration: 24th of July 2023.

## Introduction

Psoriasis is a chronic systemic inflammatory disease associated with various metabolic disorders such as obesity, especially visceral obesity, dyslipidemia, insulin resistance, hypertension and liver steatosis [1–3]. The systemic inflammation caused by psoriasis and the metabolic disorders that are often present in most psoriasis patients increase their cardiovascular risk [4]. Furthermore, metabolic disorders, such as obesity and insulin resistance not only exacerbate the severity of psoriasis, but can also trigger its onset [5, 6]. As cardiovascular disease is the leading cause of morbidity and mortality in psoriasis patients, reducing cardiovascular risk is of critical importance [7].

Biologic therapy significantly reduces skin lesions in many psoriasis patients by targeting a specific cytokine [2, 8]. The use of biologics is constantly increasing, as is the proportion of young psoriasis patients [9]. Since biologic therapy has a systemic effect, one might expect it to also improve or even normalize metabolic disorders by reducing systemic inflammation. However, there are few studies on this topic, especially on newer biologic treatments, such as inhibitors of interleukin (IL)-17 and inhibitors of IL-23 [10–12]. In addition, studies comparing the effect of more than two different antipsoriatic therapies on metabolic disorders are lacking. Therefore, it is of crucial clinical importance to determine whether and to what extent metabolic disturbances are present in the stable state of psoriasis patients successfully treated with different biologics.

The aim of the present study was to investigate metabolic disturbances in (a) patients treated with three different biologic therapies (TNF-α inhibitor, anti-IL-17 agent and anti-IL-23 agent), (b) patients treated with methotrexate and (c) patients treated with a topical treatment and to compare them between the different treatment groups and with apparently healthy, age-matched controls. To avoid potential confounders, only young to middle-aged patients without additional comorbidities and without additional medications were included in this study. In addition, only patients with successful skin clearance were included to reflect the real-life clinical scenario in which biologic therapy is completely successful and the inflammatory burden is alleviated. Patients with chronic and stable state of psoriasis treatment were selected to investigate the stable condition of the patients.

## Materials and methods

### Study population and design

We conducted a cross-sectional study of 80 patients (54 men and 26 women) with psoriasis and 20 healthy age and sex matched participants (11 men and 9 women) at the Dermatology Outpatient Clinic, Department of Dermatovenerology, University Medical Centre Ljubljana, Ljubljana, Slovenia. We recruited consecutive patients who had successfully treated psoriasis with topical therapy (*n* = 21), methotrexate (*n* = 11), adalimumab (*n* = 14), secukinumab (*n* = 14) or guselkumab (*n* = 20). The patients received no treatment other than antipsoriatic treatment. The efficacy of the treatment was defined by the Psoriasis Area Severity Score (PASI). Treatment was rated excellent in 99% of patients (PASI < 1) and good in 1% of patients (PASI 1–3). All participants met the following inclusion criteria: diagnosis of psoriasis, age between 30 and 45 years, successful treatment with topical therapy, methotrexate, adalimumab, secukinumab or guselkumab, and stable psoriasis for at least 6 months. Both patients and physicians had to be satisfied with the response to treatment and did not plan to change treatment. Exclusion criteria were previous cardiovascular events, type 1 or type 2 diabetes, menopause, pregnancy or breastfeeding, psoriatic arthritis or other chronic inflammatory diseases, and other treatments in addition to psoriasis treatment. Healthy control subjects aged 30–45 years were included with the same exclusion criteria. All participants voluntarily participated in this study and gave their informed consent. The study was approved by the Slovenian National Medical Ethics Committee (approval number 0120–422/2021/6). All methods were carried out in accordance with the Declaration of Helsinki. The study is registered at http://clinicaltrials.gov (ClinicalTrials.gov Identifier: NCT05957120). The report of this study is consistent with the STROBE guidelines [13].

### Study protocol

A complete medical history was obtained, and a complete medical examination of each patient was performed. Anthropometric measurements (weight, height, and waist circumference), systolic and diastolic blood pressure, and heart rate were obtained for each patient. Blood was collected from the antecubital vein of each patient according to the standard procedure and collected in vacuum tubes. The lipid panel (total cholesterol, low-density lipoprotein cholesterol (LDL), high-density lipoprotein cholesterol (HDL) and triglycerides) and different metabolic indices (body mass index (BMI), glycated hemoglobin (HbA1c), homeostatic model assessment for insulin resistance (HOMA-IR), triglyceride glucose index (TyG index), and fibrosis-4 index for liver fibrosis (FIB-4)) were also measured and/or calculated.

### Laboratory methods

Fasting blood samples (4 ml) were taken from each participant by venipuncture using disposable needles and syringes. After centrifugation of blood samples, serum was separated and stored at -80 °C until analysis of biochemical parameters. Serum levels of fasting blood glucose, insulin, total cholesterol, triglycerides, LDL, and HDL were measured using standard colorimetric methods. Serum levels of alanine aminotransferase (ALT) and aspartate aminotransferase (AST) were measured using kinetic methods. All biochemical analyses were performed using a semi-automated biochemical analyzer (Humalyzer 3000, USA). Glycated hemoglobin (HbA1c) was determined using the VITRO 5.1FS Chemistry System (Ortho Clinical Diagnostics, Raritan, New Jersey). All analyses were performed according to the manufacturer’s instructions. The interlaboratory precision and accuracy of all measurements were confirmed by regular calibration with the reference standards included in the kits.

### Calculation of metabolism indices

The body mass index was calculated by dividing body weight by the squared degree of body height. HOMA-IR was calculated using the following equation *(insulin (mU/L) × glucose (mmol/L)) / 22.5*. The TyG index was calculated using the equation *ln [fasting triglyceride (mg/dl) × fasting glucose (mg/dl)] /2*. FIB-4 was calculated using the equation *(age × AST (U/L)) / (platelets × √(ALT(U/L))).*

### Statistical analysis

Statistical analyses were carried out using R software (version 4.2.2) and IBM SPSS Statistics 28. Given the non-normal distribution of the data and the presence of outliers, patient characteristics were described using group medians and interquartile ranges. The nonparametric Kruskal-Wallis test was used to test the null hypothesis that the population medians of all groups are equal. If the null hypothesis was rejected, the Dunn multiple comparison test with Bonferroni correction was used to determine which specific median pairs differ significantly. Structural percentages were reported for two categorical variables, and Fisher’s exact test was also applied.

To determine whether specific metabolic parameters varied by type of psoriasis treatment, Quade’s non-parametric ANCOVA test was used, with systolic blood pressure and age incorporated as covariates in the model. In cases where the ANCOVA results were significant, pairwise comparisons were performed using Fisher’s least significant difference (LSD) method. The Benjamini-Hochberg correction was then applied to adequately control the type I error rate. The Spearman correlation coefficients were calculated to gain better insight into the parameters observed in the patient and control group.

## Results

### Patient characteristics

The characteristics of the patients are presented in Table 1. The patient groups and the control group were homogeneous. There was a statistically significant difference in the duration of psoriasis and the duration of treatment that nevertheless resulted in a good response according to PASI and BSA. The latter were statistically significantly different between the groups, but in terms of numbers, all of them meant an excellent treatment effect.

**Table 1:** Patient characteristics

	Type of treatment							
Patients’ characteristics	CG	TOP	MTX	ADA	SEC	GUS		Test statistic *p*-value
Average age (years)	34.50 (31.25- 39.75)	38.00 (32.00-41.50)	39.00 (35.00–42.00)	39.50 (36.75-41.00)	39.50 (34.50-43.25)	40.00 (36.00–43.00)		H = 9.312 *P* = 0.097
Gender Male Female	12	13	7	10	11	13		Exact test *P* = 0.882
Female	8	8	4	4	3	7		
Smokers No. (%)	6 (30)	7 (33)	3 (27)	7 (50)	8 (57)	4 (20)		Exact test *P* = 0.242
Duration of psoriasis (years)	/	8.0 (4.5–20.0)	10.0 (5.0–12.0)	20.0 (11.5–25.0)	16.50 (13.8–23.3)	20.0 (15.0-23.5)		H = 16.646 *P* = 0.002
Duration of treatment (months)	/	77.0 (47.0-239.0)	29.0 (21.0–47.0)	95.0 (61.0-117.5)	48.0 (33.5–59.0)	31.0 (27.3–51.0)		H = 31.059 *P* < 0.001
PASI	/	0.2 (0.1–1.8)	1.2 (0.7–3.2)	0.0 (0.0-0.2)	0.0 (0.0-0.7)	0.0 (0.0-0.6)		H = 17.393 *P* = 0.002
BSA (m^2^)	/	1.0 (1.0-1.5)	2.0 (1.0–4.0)	0.0 (0.0-0.3)	0.0 (0.0–1.0)	0.0 (0.0–1.0)		H = 20.849 *P* < 0.001
Systolic BP (mmHg)	127.00 (116.00-138.00)	124.00 (110.00-132.50)	137.00 (129.00-142.00)	121.00 (110.25-133.25)	123.00 (118.00-133.25)	120.00 (110.75-136.75)		H = 8.948 *P* = 0.111
Diastolic BP (mmHg)	84.50 (77.25- 90.00)	79.00 (73.00-84.50)	83.00 (80.00–95.00)	81.50 (72.00-92.25)	83.50 (81.00-93.25)	84.50 (74.00-96.75)		H = 7.084 *P* = 0.214

Data are presented as median (interquartile range), or number of cases for categorical variables. The P-value in the last column was determined using the Kruskal-Wallis H test, ANOVA or Fisher-Freeman-Halton exact test. CG, control group; TOP, topical therpay; MTX, methotrexate; ADA, adalimumab; SEC, secukinumab; GUS, guselkumab; PASI, Psoriasis Area and Severity Index, BSA, Body Surface Area, BP, blood pressure.

### Specific metabolic parameters in five groups of patients and controls

**Fig. 1:**
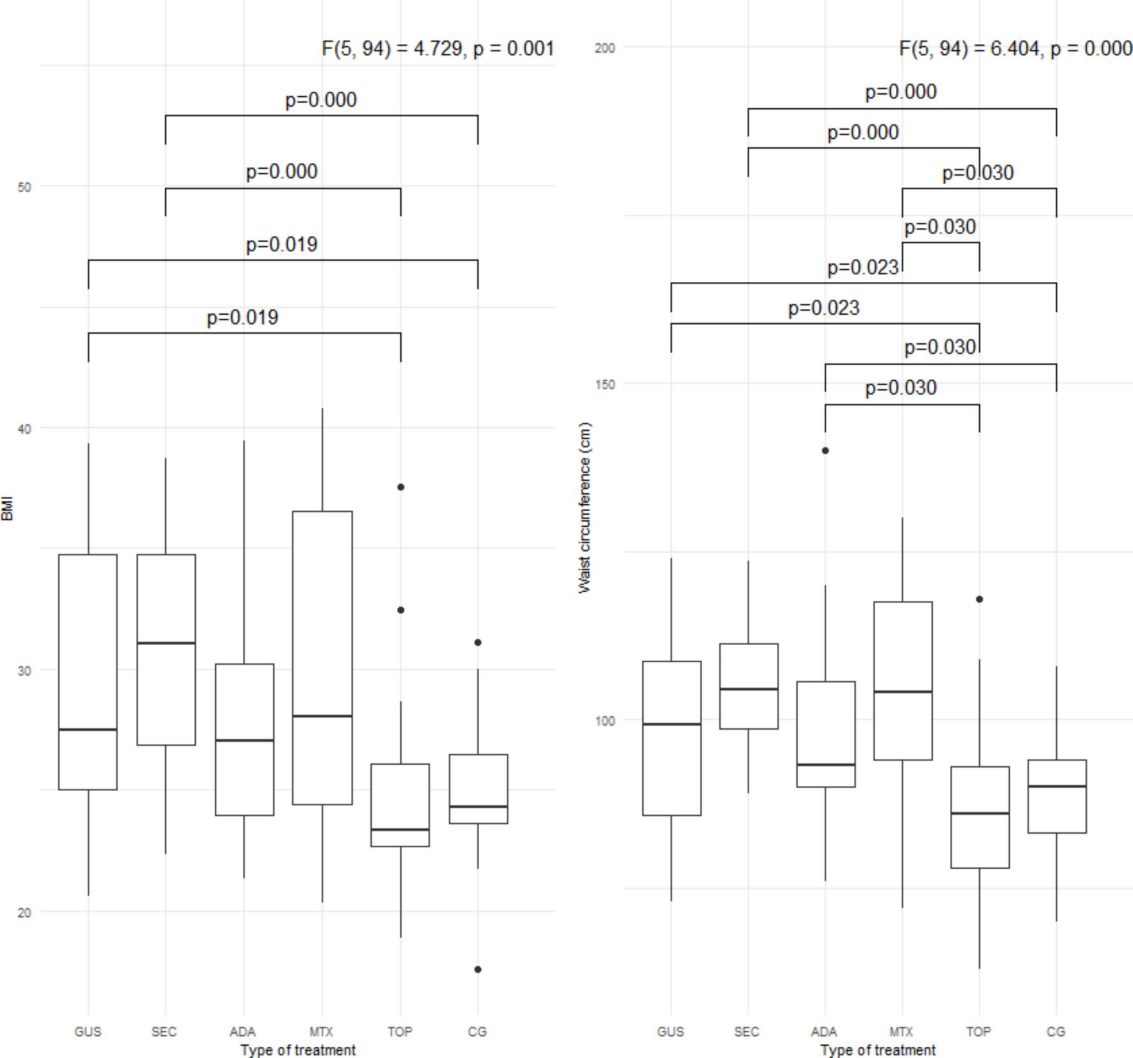
Body mass index (BMI) and waist circumference in the 5 groups of psoriasis patients and the control group. BMI, body mass index; GUS, guselkumab; SEC, secukinumab; ADA, adalimumab; MTX, methotrexate; TOP, topical therapy; CG, control group. *Quade’s ANCOVA and LSD post hoc test with Benjamini-Hochberg correction was applied*,* with only statistically significant pairs shown in the figure *(*P* ≤ *0.05*)

**Fig. 2:**
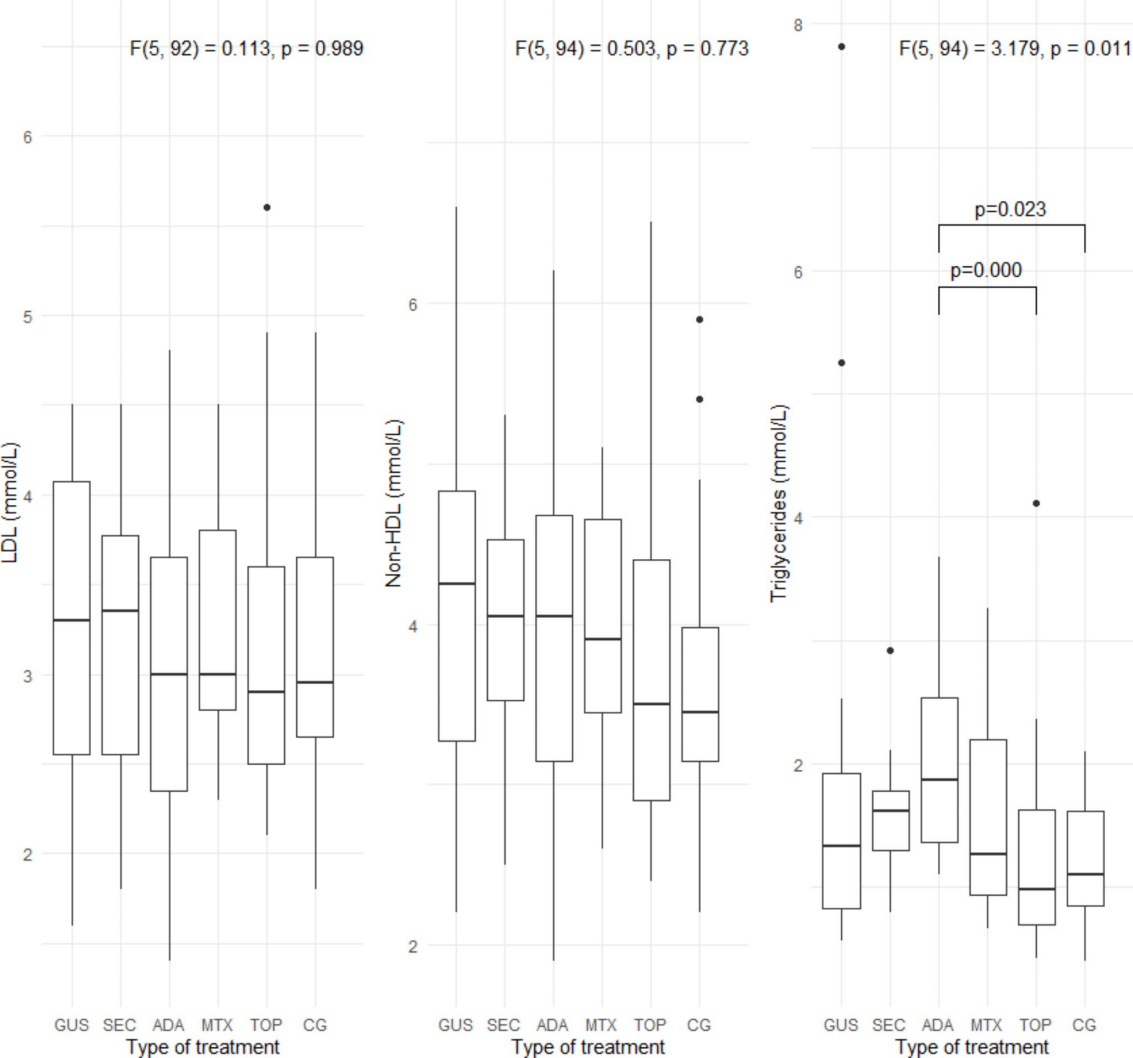
LDL, non-HDL, and triglyceride levels in all 5 groups of psoriasis patients and the control group. LDL, low-density lipoprotein; non-HDL, non-high-density lipoprotein; GUS, guselkumab; SEC, secukinumab; ADA, adalimumab; MTX, methotrexate; TOP, topical therapy; CG, control group. *Quade’s ANCOVA and LSD post hoc test with Benjamini-Hochberg correction was applied*,* with only statistically significant pairs shown in the figure *(*P* ≤ *0.05*)

**Fig. 3:**
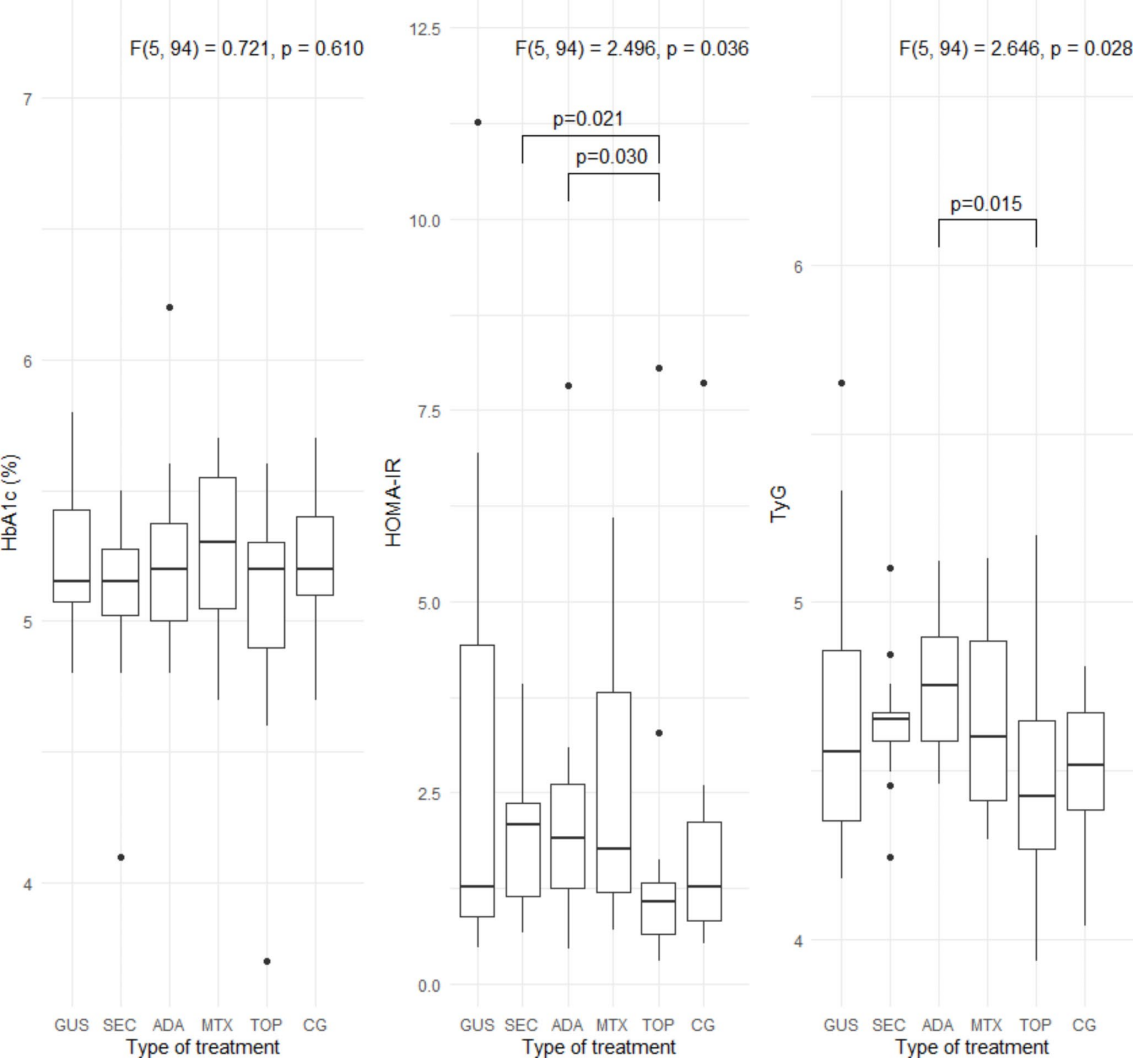
Insulin resistance parameters (HbA1c, HOMA-IR, and TyG index) in the 5 groups of patients with psoriasis and in the control group. HbA1c, glycated hemoglobin; HOMA-IR, homeostatic model assessment for insulin resistance; TyG, triglyceride-glucose index; GUS, guselkumab; SEC, secukinumab; ADA, adalimumab; MTX, methotrexate; TOP, topical therapy; CG, control group. *Quade’s ANCOVA and LSD post hoc test with Benjamini-Hochberg correction was applied*,* with only statistically significant pairs shown in the figure *(*P* ≤ *0.05*)

**Fig. 4:**
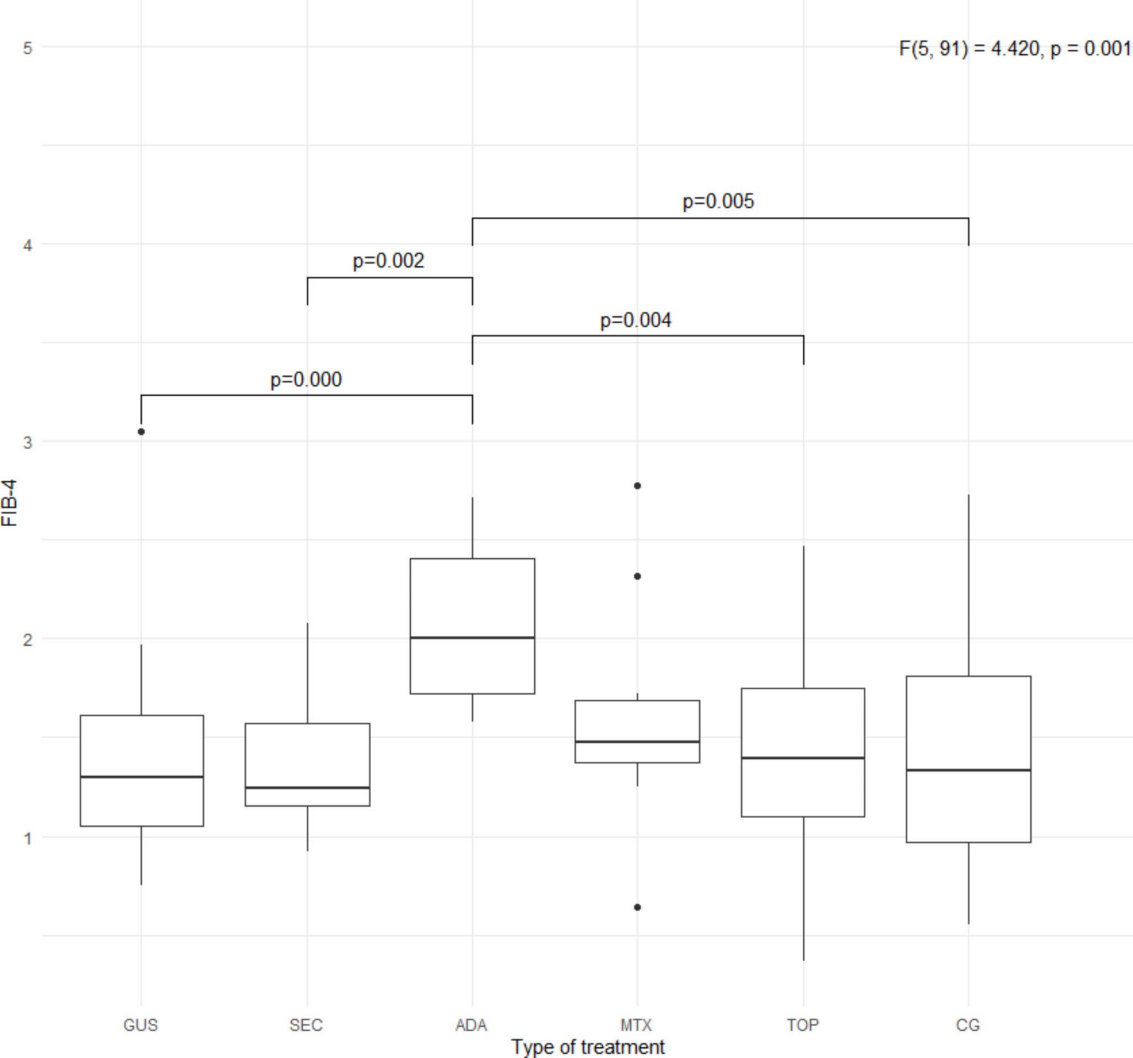
FIB-4 index in the 5 groups of patients with psoriasis and in the control group. FIB-4, fibrosis-4 index; GUS, guselkumab; SEC, secukinumab; ADA, adalimumab; MTX, methotrexate; TOP, topical therapy; CG, control group. *Quade’s ANCOVA and LSD post hoc test with Benjamini-Hochberg correction was applied*,* with only statistically significant pairs shown in the figure *(*P* ≤ *0.05*)

**Fig. 5:**
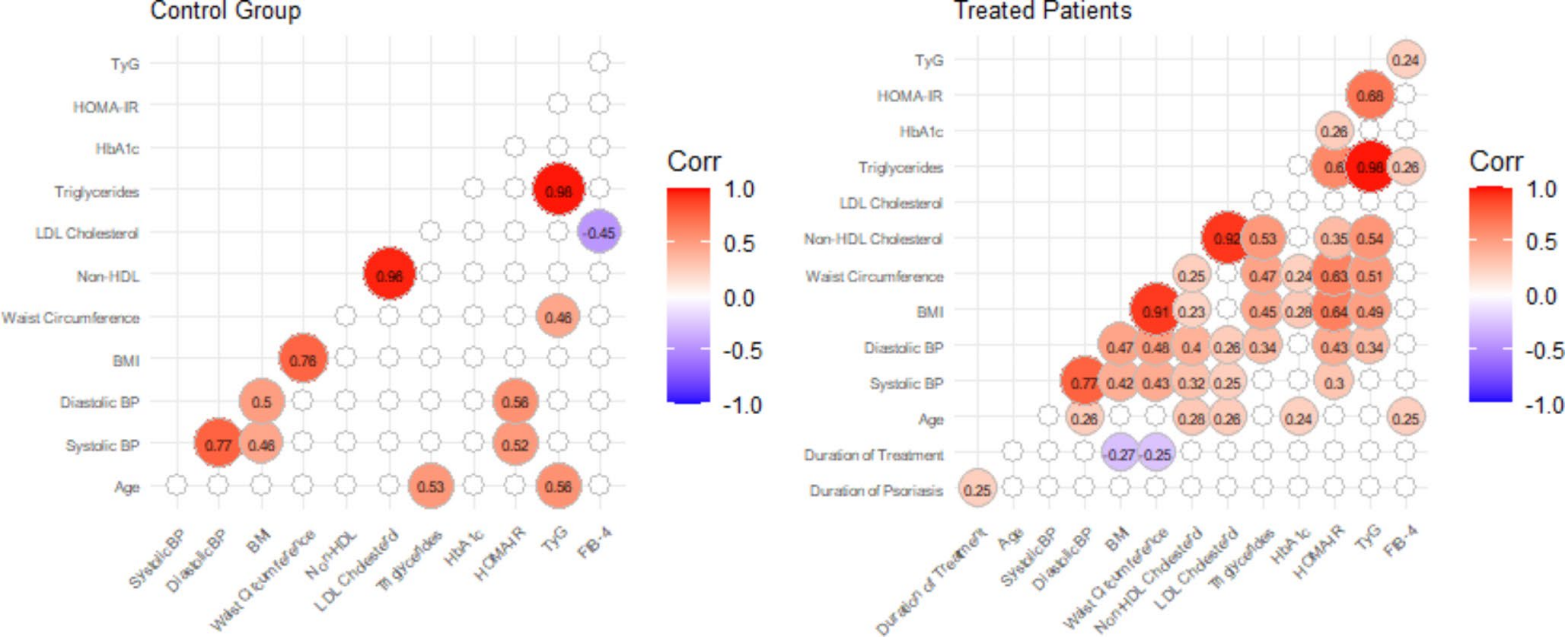
Correlation matrix with Spearman’s correlation coefficients for the control group and for the treated patients, showing only significant correlations (*P* ≤ 0.05). TyG, triglyceride-glucose index; HOMA-IR, homeostatic model assessment for insulin resistance; HbA1c, glycated hemoglobin; LDL, low-density lipoprotein; HDL, high-density lipoprotein; BMI, body mass index; BP, blood pressure

Specific metabolic parameters (BMI, waist circumference, LDL, non-HDL, triglyceride levels, HbA1c, HOMA-IR, TyG index, and FIB-4 index) are shown in Figs. 1, 2, 3 and 4. Patients treated with guselkumab and secukinumab had a higher BMI and waist circumference than the topical treatment group or the control group. Similarly, in the adalimumab and methotrexate groups, waist circumference was significantly higher than in the topical treatment and control groups. There were no statistically significant differences in LDL, non-HDL, and HbA1c, in the 5 psoriasis treated groups compared to each other and compared to the control group (Figs. 2 and 3). Patients treated with adalimumab showed a statistically significant elevated triglyceride level, HOMA-IR, and TyG index compared to those treated with local therapy (Figs. 2 and 4). However, no consistent significant differences were found between the three groups treated with biologic therapy (Figs. 1, 2 and 3). Patients treated with adalimumab had significantly higher FIB-4 index than those treated with secukinumab and guselkumab, as well as higher than patients treated with local therapy or controls. In the patient groups, there were several statistically significant correlations (Fig. 5). All of these correlations can be explained in accordance with a metabolic syndrome that comprise abdominal obesity (BMI, waist circumference), arterial hypertension, dyslipidaemia, and insulin resistance. In the control group, these correlations were not present (Fig. 5).

## Discussion

The results of our study indicate that young patients with psoriasis who were successfully treated with three different biologics have a similar metabolic status to the control group. Our results showed no relevant differences compared to controls in the parameters of dyslipidemia and insulin resistance and some differences in triglyceride levels and FIB-4 index, which however did not consistently show a similar trend for all three biologics. In addition, there were no differences between all three biologics. Therefore, our results suggest that there is no significant residual metabolic burden in patients successfully treated with biologics. This underscores the importance of successful treatment in the management of psoriasis.

We studied and compared six groups of patients (three groups treated with biologics, two treated with methotrexate or topical treatment and one control group). Importantly, all patients were successfully treated (PASI score less than 1 in 99% of patients), were in the stable phase of the disease and did not receive any other treatment. Thus, we measured specific metabolic parameters in a stable disease stage in which neither the patients nor the control group had any other concomitant diseases or treatments in addition to psoriasis treatment. To exclude confounding factors that could influence metabolic status, such as comorbidities and age, we included young patients without other chronic diseases. Most published studies examined only one or two treatment modalities, whereas we examined and compared five treatment modalities that are most commonly used in the treatment of psoriasis. In addition, most previous studies have examined the effects of treatment on blood glucose levels, blood lipids and body composition parameters [10, 14–16], while data on clinically useful markers of metabolic status such as HOMA-IR, TyG index and FIB-4 index are sparse despite their usefulness as markers for the detection of metabolic disorders [17–19]. In addition, the TyG index appears to be useful for the detection of subclinical atherosclerosis in patients with psoriasis [19, 20].

Several studies have been published investigating the effects of biologics on metabolic parameters. TNF-α inhibitors have shown beneficial effects on blood lipid and blood glucose levels [15, 16]. A post-hoc analysis showed a neutral effect of secukinumab on fasting plasma glucose, lipid parameters and liver enzymes [10], while another study with secukinumab and ixekizumab showed a reduction in LDL levels and an improvement in HDL levels, as well as mixed results on body composition parameters [16, 21]. In a more recent study of the following biologic therapies, ixekizumab, secukinumab, guselkumab, certolizumab, ustekinumab, risankizumab and adalimumab, researchers observed a significant reduction in metabolic dysfunction following biologic treatment [22]. However, 56.6% of patients had metabolic syndrome at baseline and the age of patients ranged from 18 to 74 years, so confounding factors could not be excluded. This study did not examine the impact of treatment efficacy, particularly the complete clearance of skin. None of the available studies specifically examined the metabolic status of successfully treated young patients compared to controls and to patients treated with methothrexate and topical treatment. As the group of young patients without comorbidities is large, it seems that such information would have relevant clinical value.

Th17 cells are critically involved in the pathogenesis of psoriasis by producing pro-inflammatory cytokines such as TNF-α, IL-17 and IL-22, which stimulate hyperproliferation and altered keratinocyte differentiation in psoriasis. These cytokines are also involved in obesity, insulin resistance, diabetes mellitus and metabolic dysfunction-associated steatotic liver disease (MASLD) [23]. In addition, TNF-α contributes directly to insulin resistance [19], while IL-17 appears to link psoriasis and hyperglycemia [24]. In addition, IL-12 and IL-23 have been linked to atherosclerosis, as both cytokines are present in atherosclerotic plaques [25]. Blocking IL-23 has been shown to significantly reduce IL-17 levels, potentially impacting metabolic disorders [26]. Therefore, inhibition of TNF-α, IL-17 and IL-23 could alleviate or protect against metabolic disorders, but the data are still limited and conflicting, but obviously needed. It seems that the appropriate approach would be to study metabolic disorders separately in different patient groups that differ according to age, comorbidities and additional pharmacological treatment. Our results show that young patients who have been successfully treated with biologics and have no comorbidities, which constitute an important part of psoriasis patients, have a similar metabolic profile to the control group and do not have additional metabolic disturbances.

Our study has several limitations. The lack of data on metabolic parameters before the start of therapy is a limitation of this cross-sectional study. However, we did not focus on metabolic changes before and after treatment, but rather on comparison with controls and other treatment modalities in the stable phase of the disease. Another limitation was the relatively small number of patients in each group. However, the similar results in three groups of patients treated with biologics strongly support the credibility of the results and conclusions.

## Conclusions

In summary, our results show that young psoriasis patients successfully treated with biologic therapy (adalimumab, secukinumab or guselkumab) had similar metabolic parameters as the control group and those treated with topical therapy or methotrexate. This indicates that there is no significant residual metabolic burden in these patients. These results are clinically relevant and should be considered in the treatment of psoriasis patients.

## Data Availability

The data used and analyzed during the current study is available from the corresponding author on reasonable request.
